# Turnout and Territorial Reform: Data from Sub municipal governments in Portugal

**DOI:** 10.1016/j.dib.2020.105598

**Published:** 2020-04-22

**Authors:** Miguel Rodrigues, António Tavares

**Affiliations:** Research Central in Political Science, School of Economics and Management, University of Minho

**Keywords:** Electoral turnout, Territorial reform, Turnout

## Abstract

This database is related to the voter turnout in five election cycles (2001, 2005, 2009, 2013 and 2017). The data was used in the original in the article “The effects of amalgamations on voter turnout: Evidence from sub-municipal governments in Portugal” (DOI: 10.1016/j.cities.2020.102685). The evolution of turnout in Portuguese sub-municipal units (SMUs) has followed the trend of western democracies and recorded a continuous drop. The 2013 territorial reform of SMUs contributed to the increment of the gap of electors that choose not to cast a vote in the elections. Data provides further details on how the territorial reform had an impact on the level of electoral participation. It also adds data on SMUs turnout divided by type and whether or not SMUs were merged.

Specifications table

**Table:** 

Subject	Sociology and Political Science
Specific subject area	Electoral participation in Sub municipal governments
Type of data	Table Graph
How data were acquired	The dataset was built with raw data from the national electoral commission concerning the electoral outcome. We match the number and codes of the SMU in order to have consistency across the electoral cycles (2001, 2005, 2009, 2013 and 2017). Data on the classification of SMU was gathered from the National Institute of Statistics.
Data format	Raw, processed and analyzed.
Parameters for data collection	This is a unique database that gathers information for turnout in the amalgamated and non-amalgamated SMU before and after the territorial reform of 20013. It has potential interest for academics willing to do research on electoral participation.
Description of data collection	The data was gathered from the national electoral commission and the National Institute of Statistics for the years of 2001, 2005, 2009, 2013 and 2017, corresponding to the political cycle of local elections for SMU. Variables in the database include the code of the SMU, number of voters in each jurisdiction, the levels of turnout recorded in each moment, the classification of the SMU (Rural vs Urban), the identification of the year and the situation towards the territorial reform (amalgamated or not).
Data source location	Research Centre in Political Science (CICP), School of Economics and Management, University of Minho, Portugal
Data accessibility	Data available online on https://data.mendeley.com/datasets/yrb99wgpsc/1 DOI: 10.17632/yrb99wgpsc.2
Related research article	Title: The Effects of Amalgamations on Voter Turnout: Evidence from Sub-Municipal Governments in Portugal. Journal: Cities DOI: 10.1016/j.cities.2020.102685

## Value of the Data

• The data provides information related to the levels of turnout in sub municipal units (SMU) from 2001 until 2017.
• The data available provides an added intel to researchers in political science that seek to analyse the behaviour of turnout in SMU;
• The division of SMU into two separated groups (amalgamated and not) allows the specification of econometric models to assess the impact of the territorial reform;
• Data available can allow the forecast and the comparability of the effect of the territorial reform and the amalgamation process across countries;

## 1 Data

The data included in the article have the information of turnout from sub-municipal units (SMU) for five electoral periods with a total of 14.663 observations. Besides the levels of turnout, the database has information about the number of registered voters, whether or not the SMU was amalgamated. Additionally, the database is divided by two dummy variables: one that identifies the SMU that was subject to the process of territorial reform; and the other, referring to time to signal if the data refers to the period before or after the territorial reform. All data is available and deposited at Mendeley (http://doi.org/10.17632/yrb99wgpsc.2) The name and measurement of the variables are as described in Table 1.

**Table 1:** Variable and description.

Variable	Description
SMU	Official code given by the National Institute of Statistics to the SMU
Registered Voter	Number of registered voters in each jurisdiction provided by the national electoral commission
Turnout	Level of turnout for each SMU provided by the national electoral commission
Treated	1 if SMU was affected by the territorial reform, 0 otherwise
Year	Indication of the year
APU	1 if SMU is classified as urban, 0 otherwise

## 2 Experimental Design, Materials, and Methods

**Fig. 1:**
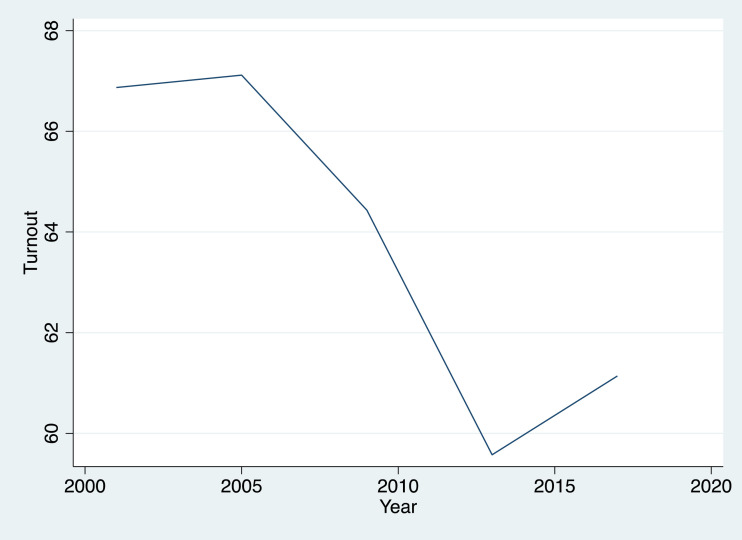
Evolution of turnout by year for all SMU.

Data in Table 1 show the evolution of the turnout in all SMU for the period of time between 2001 and 2017 – that is, five electoral moments. As we can see in Fig. 1, the overall evolution of turnout is quite negative. Since 2005 until 2013, the data show a significant drop in the level of turnout. Although some recovery can be seen in the latest election, it didn't compensate for the previous drop.

**Fig. 2:**
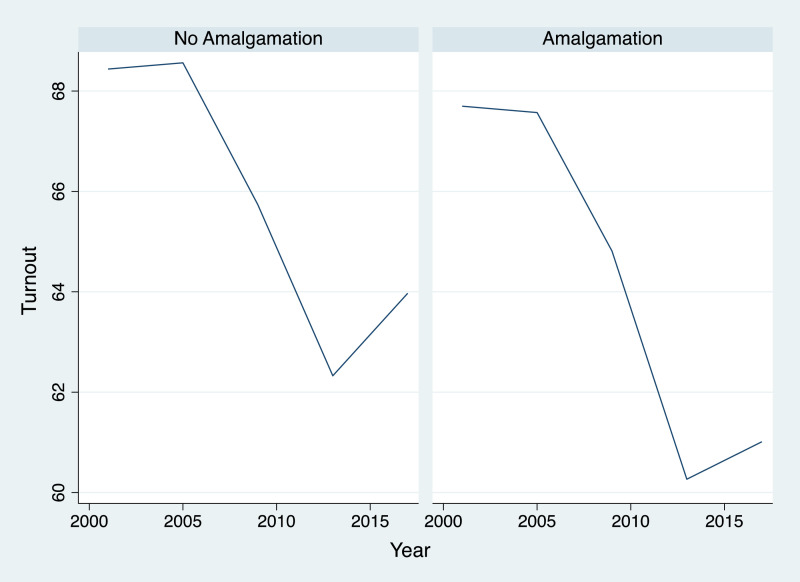
Evolution of turnout for SMU affected, and not, by the territorial reform.

If we opt to segregate data between the SMU that were affected by the territorial reform, we can identify (Table 3) a different pattern. The jurisdictions that went through the process of mergers experience an extra drop in their levels of turnout. The fact is that, even before the territorial reform, we can notice a difference between jurisdiction. Amalgamated SMU already recorded lower levels of turnout. However, the drop that we noticed on the overall electoral participation (Fig. 1) is much more pronounced in amalgamated units (Fig. 2).

We can also divide the data taking into account the classification of the SMU (Urban or rural). First, data in Table 3 show that urban SMU, traditionally, records a lower level of turnout. The highest level of turnout in urban SMU barely overcomes the lowest level of turnout in rural SMU.

**Fig. 3:**
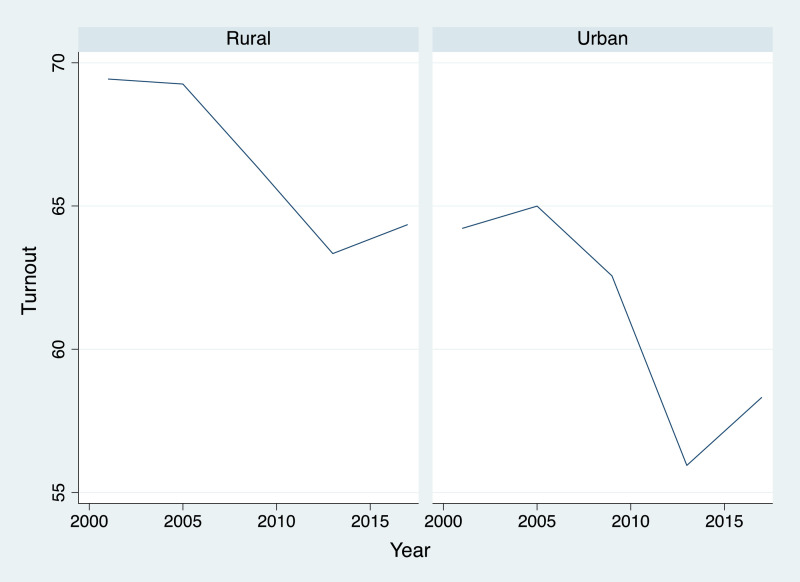
Evolution of turnout for different types of SMU.

Fig. 3 explicitly show the differences between the two kinds of SMU. Taking into account the drop in turnout of 2013 in rural SMU, we needed to go back to 2005 figures for urban SMU, to find similar levels.

We use panel data analysis with fixed effects to capture the effect of the specific attributes of SMUs that could be decisive in the selection of a SMU to be merged (treatment group). The model can be formulated as follows:

$$Turnout_{i,t}=\beta_{1}+\beta_{2}Treated_{i,t}+\beta_{3}Year_{i,t}+\beta_{4}Size_{i,t}+i\alpha_{i,t}+\varepsilon_{i,t},$$

We computed a model in which the dependent variable is the level of turnout in each of the five election cycles in our analysis (2001, 2005, 2009, 2013 and 2017). We use a dummy variable to identify the treated parishes after the treatment (Treated), which enables us to assess the effect of the territorial reform. This variable captures the effect in turnout of the amalgamation process. We also added another dummy variable to control for the years after the territorial reform (Year). This variable will capture the variation in turnout in the years after the territorial reform for all parishes regardless of whether they were affected by the amalgamation. Additionally, the number of registered voters (per thousand) was also added to control for the selection criteria used by the UTRAT. The unknown fixed effect for each observation is gauged by the term i, which controls all the unobserved attributes of the parishes that may have affected their inclusion in the Treated group.

The results from the panel data estimation show that the territorial reform has a negative impact on electoral participation since merged SMUs have lower turnout levels (Table 5).

The dummy for the Year is negative, meaning that, on average, we find a negative variation of 4.43 percentage points between the election before the territorial reform and the one after it. The variable controlling for the selection criteria of the parishes to be merged (Registered Voters) displays a negative sign of 0.04 percentage points but is not significative. This leaves the results of the variable Treated with a more reliable dimension of the effect of the merger on the level of voter turnout of the merged SMU. Again, we stress that this dummy variable takes the value of one to identify the merged SMU after the treatment and assumes zero in all the remaining situations. The results show a negative impact of the mergers on turnout levels: the level of voter turnout is lower by 1.462 percentage points than if they had not been subjected to the amalgamation reform (Tables 2 and 4).

**Table 2:** Levels of turnout by year for all SMU.

Year	Turnout
2001	68.2%
2005	68.25%
2009	64.45%
2013	61.6%
2017	62.94%

**Table 3:** Levels of turnout by year and effect of the territorial reform for all SMU.

Year	Amalgamation	Turnout
2001	Yes	67.70%
	No	68.44%
2005	Yes	67.57%
	No	68.56%
2009	Yes	64.81%
	No	65.74%
2013	Yes	60.26%
	No	62.32%
2017	Yes	61.01%
	No	63.97%

**Table 4:** Levels of turnout by year and SMU classification.

Year	SMU Classification	Turnout
2001	Rural	69.43%
	Urban	64.22%
2005	Rural	69.26%
	Urban	64.99%
2009	Rural	66.34%
	Urban	62.56%
2013	Rural	63.34%
	Urban	55.95%
2017	Rural	64.35%
	Urban	58.32%

**Table 5:** Results of the Panel Data Analysis with fixed effects.

	2001-2017
$Treated_{i,t-1}$	-1.462***
	(0.194)
*Year*_*i, t*_	-4.43***
	(0.097)
*Size*_*i, t*_	-0.04
	(0.058)
Constant	67.451***
	(0.204)
N	14,645
Groups	3,023
*Prob* > *F*	0.000
type="Other"Rho	0.774

*p* < 0.1; ** *p* < 0.05; *** *p* < 0.01

